# Autologous Hematopoietic Stem Cell Transplantation in Multiple Sclerosis Patients: Monocentric Case Series and Systematic Review of the Literature

**DOI:** 10.3390/jcm11040942

**Published:** 2022-02-11

**Authors:** Francesco Patti, Clara Grazia Chisari, Simona Toscano, Sebastiano Arena, Chiara Finocchiaro, Vincenzo Cimino, Giuseppe Milone

**Affiliations:** 1 Department of Medical, Surgical Sciences and Advanced Technologies “G.F. Ingrassia”, University of Catania, 95125 Catania, Italy; simonatoscano@hotmail.it (S.T.); sebarena780@gmail.com (S.A.); chiara.finocchiaro@hotmail.it (C.F.); 2 IRCCS Centro Neurolesi “Bonino Pulejo”, 98124 Messina, Italy; vincenzo.cimino@irccsme.it; 3 Hematology and Bone Marrow Transplant Unit, Azienda Policlinico-Vittorio Emanuele, 95124 Catania, Italy; giuseppe.milone@gmail.com

**Keywords:** multiple sclerosis, treatment, hematopoietic stem cell transplantation

## Abstract

Multiple sclerosis (MS) is a chronic, inflammatory and immune-mediated disease of the central nervous system (CNS), commonly affecting young adults and potentially associated with life-long disability. About 14 disease-modifying treatments (DMTs) are currently approved for the treatment of MS. However, despite the use of highly effective therapies, some patients exhibit a highly active disease with an aggressive course from onset and a higher risk of long-term disability accrual. In the last few years, several retrospective studies, clinical trials, meta-analyses and systematic reviews have investigated autologous hematopoietic stem cell transplantation (AHSCT) as a possible therapeutic option in order to address this unmet clinical need. These studies demonstrated that AHSCT is a highly efficacious and relatively safe therapeutic option for the treatment of highly active MS. Particularly, over recent years, the amount of evidence has grown, with significant improvements in the development of patient selection criteria, choice of the most suitable transplant technique and clinical experience. In this paper, we present six patients who received AHSCT in our MS center and we systematically reviewed recent evidence about the long-term efficacy and safety of AHSCT and the placement of AHSCT in the rapidly evolving therapeutic armamentarium for MS.

## 1. Introduction

Multiple sclerosis (MS) is a chronic, inflammatory, neurodegenerative and immune-mediated disease of the central nervous system (CNS) characterized by inflammatory demyelination and subsequently gliosis and axonal loss [1,2].

Over the last two decades, thanks to the growing knowledge about MS pathogenesis, a fair amount of disease-modifying therapies (DMTs) have been developed for the treatment of MS. These drugs have different mechanisms of action directed to control the inflammatory component of the disease, with the result of reducing the risk of relapse and the accumulation of the disability [2]. To date, a total of 14 DMTs have been approved with different efficacies and safety profiles. Despite their use, many patients remain at some risk for relapses and disability progression; in particular, those exhibiting a severe disease activity despite the use of highly active treatment or showing an aggressive course from disease onset are exposed to a high risk of long-term disability accrual [3]. In these cases, autologous hematopoietic stem cell transplantation (AHSCT) can be considered as a possible therapeutic option. AHSCT has been employed to treat MS over the last two decades and, unlike most of the currently approved DMTs, it was originally applied in hematologic settings and, since the 1990s, has been used to treat refractory autoimmune diseases [4]. 

The aim of AHSCT is to remove the aberrant immune system and then to reconstitute the immune system in order to prevent recurrent neuroinflammatory activity [5,6]. In contrast with currently approved DMTs, AHSCT is an intensive one-off treatment, following which, most of the patients will have no need for additional therapies. Indeed, AHSCT provides a rapid resolution of disease activity, either in terms of relapse or disability progression, while, in the long term, the reset immune tolerance due to the reconstitution of a new immune system is associated with sustained clinical responses.

In the past, concerns regarding the mortality rate of AHSCT have previously limited its use for the treatment of MS, as MS is usually a non-life-threatening disease. However, thanks to the increasing transplant center experience and careful patient selection over the few last years, AHSCT has become relatively safe with minimal risk of treatment-related mortality. For all these reasons, in October 2020, the National Multiple Sclerosis Society proposed AHSCT as a suitable treatment option for relapsing MS patients who exhibit higher disease activity in terms of a high relapse rate and/or new/enlarged MRI lesions, despite treatment with second-line DMTs or have contraindications to highly effective DMTs [7]. 

In the present paper, we present six cases of MS who received AHSCT in our MS center and we reviewed the main findings about the efficacy and safety of AHSCT for the treatment of MS patients and the new placement of AHSCT in the rapidly evolving therapeutic scenario of MS.

## 2. Materials and Methods

### 2.1. Case Series Presentation

In this study, we collected data about 6 patients who had received AHSCT for MS in our MS center in the period between 2019 and 2021. All patients received AHSCT in the Hematology Department at the Hospital “G. Rodolico—San Marco”, University of Catania (Catania, Italy). 

### 2.2. Literature Search

**Figure 1:**
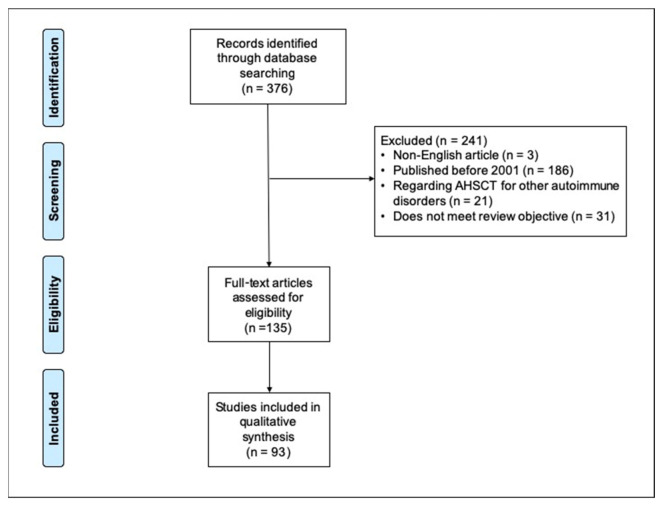
PRISMA flow. AHSCT, autologous hematopoietic stem cell transplantation.

We systematically searched all the published studies reporting AHSCT for MS on PubMed, Embase, Web of Science, Cochrane and Clinical Trials. The literature review was carried out using keywords and MeSH terms for the concepts “multiple sclerosis” and “autologous hematopoietic stem cell transplantation”. We included all the peer-reviewed, full-text and English language articles published between 2001 and 2021. In particular, randomized controlled trials (RCTs), longitudinal studies, non-randomized clinical trials, retrospective studies, meta-analyses, reviews and studies from national and international registries were selected. We excluded single case studies, pediatric studies and non-peer-reviewed papers. If publications were not available via open or institutional access, the authors of the papers were contacted. The PRISMA flow diagram is illustrated in Figure 1.

## 3. Case Series

Six patients, including three (50%) females, with a mean age of 35.8 ± 10.9 years, received AHSCT for MS in our MS center (Table 1). Three of them were affected by highly active RMS disease and the remaining had SPMS. The mean follow-up was 16.6 ± 9.8 months. All patients underwent a BEAM+ATG conditioning regimen. After the procedure, our cohort showed that the relapse rate was considerably reduced in all patients, with no relapses occurring after AHSCT. In our cohort, the Expanded Disability Status Scale (EDSS) improved in three out of six patients after AHSCT, stabilized in two and worse in only one after one year, confirming the high efficacy of AHSCT in terms of EDSS stabilization. In contrast, the only patient who reported disease progression had a higher level of disability at the time of the transplant procedure. 

The risk profile of AHSCT was acceptable and the grade of AEs (in three patients) was mild. No severe AEs were reported. So far, no long-term toxicity has been reported.

Moreover, all patients were vaccinated with both doses of the Comirnaty vaccine (BioNTech, Pfizer^®^, Mainz, Germany) and four of them underwent the booster dose. A measurement of SARS-CoV-2 antibodies performed after the vaccination showed a slightly lower response compared to the general population. 

## 4. Biological Background and Rationale

The rationale for using AHSCT as a treatment option for MS is the removal of the self-reactive immune system through immunoablative agents, resulting in the depletion of autoreactive effector cells and the reconstitution of an immune system with more normal functions thanks to the infusion of autologous hematopoietic stem cells [8,9]. After AHSCT, the analysis of circulating lymphocytes shows the decrease in the circulating autoreactive effector T cells, mostly Th17, together with the development of recent thymic emigrants post transplant [4,10,11]. The grade of the variability of the reconstituted T cell depends on the intensity of the conditioning regimen [12]. According to a recent study, prior to the transplant procedure, it was demonstrated that the presence of circulating mucosal-associated invariant T cells (MAITs) with CD8+, a CD161 high phenotype [13], may play a role in the pathogenesis of MS, promoting the production of several cytokines, such as interferon-gamma and interleukin-17a, and thus, exerting a pro-inflammatory effect [14]. After transplantation, these cells showed a significant reduction in the peripheral blood, together with an increment in regulatory CD4+, FoxP3+ T cells, CD127 and CD25 high [15]. The reconstitution of this “new” immune system may be responsible for the high efficacy of AHSCT, which is sustained even in the case of the re-occurrence of autoreactive effector cells [10]. In addition, other mechanisms were proposed, including changes in the genes expression, in particular, the downregulation of microRNAs associated with MS pathogenesis [11,16]. Thus, the complex mechanisms responsible for the long-term benefit of AHSCT are not fully understood.

## 5. Procedure

**Figure 2:**
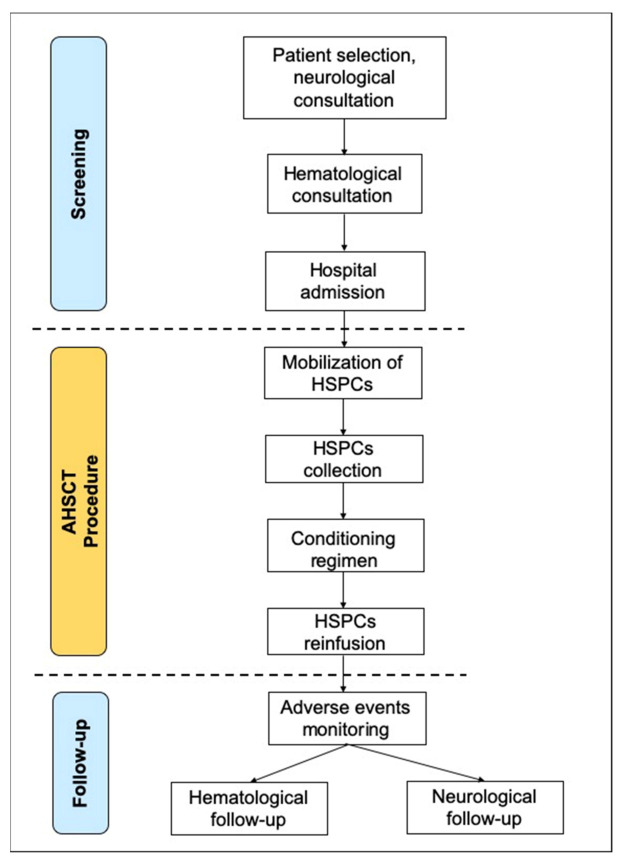
AHSCT procedure.

AHSCT consists of four main steps: mobilization of hematopoietic stem/progenitor cell (HSPC), HSPs collection, conditioning and reinfusion of HSPs and blood/immune therapy [17]. The procedure should be performed in a hematological ward with accredited stem cell laboratory facilities associated with stem cell transplant programs (Figure 2).

After neurological and hematological consultations aimed at verifying the inclusion criteria and discussing the benefits and the risks of the autologous hematopoietic stem cell transplantation (AHSCT) procedure, the MS patient is admitted to a hematological ward (screening). This first step includes mobilization consisting of the administration of chemotherapy, mainly cyclophosphamide (Cy) with a dosage of 2–4 g/m^2^, followed by daily granulocyte-colony stimulating factor (G-CSF) with the dosage of 5–10 μg/kg daily until harvest completion. Following mobilization into the circulation and after an average of 10 days, hematopoietic stem/progenitor cells (HSPCs) are collected through leuko-apheresis. The autoreactive immune cells are systematically depleted from the bone marrow, peripheral blood, lymphoid tissue and the CNS through the infusion of several immunodepleting drugs (conditioning regimen). According to the European Society for Blood and Marrow Transplantation (EBMT) guidelines, there are three grades of intensity of conditioning regimens (high, intermediate and low-intensity protocols), according to the degree of myelotoxicity and immunodepletion. Then, HSPs are re-infused together via hydration, antiemetics and other supportive care measures reducing the effects of the dimethyl-sulfoxide and cytolysis debris. The engraftment typically occurs on average within 10–15 days. Then, the patient can be discharged and monitored for potential late adverse events (follow-up). 

### 5.1. Mobilisation of HSPs

Mobilization is usually realized through the administration of chemotherapy, mainly cyclophosphamide (Cy) with the dosage of 2–4 g/m^2^, followed by daily granulocyte-colony stimulating factor (G-CSF) with the dosage of 5–10 μg/kg daily, until harvest completion. As an alternative, although G-CSF could be used alone, the combination with priming chemotherapy should be favored as it is associated with a lower risk of disease flares and the percentage of potential auto-reactive cells in the graft [18]. Cy or steroids may also prevent a possible exacerbation of MS induced by the G-CSF and allow for more effective mobilization, resulting in a lower burden of lymphocytes in the graft and better control of the disease [18].

### 5.2. HSPs Collection

Following mobilization into the circulation and after an average of 10 days, HSPs are collected through leuko-apheresis. The optimal target is about 5 × 10^6^/kg of body weight with a minimum safety threshold of 2 × 10^6^/kg [17]. The HSPs are collected through multiple aspirations from the bone marrow of the iliac crests or via apheresis from peripheral blood after mobilization into the circulation [19]. 

HSPs and progenitor cells mobilized into the bloodstream represent the most frequently used resource for MS patients receiving HSCT. Indeed, the higher proportion of progenitor cells and the more rapid engraftment may reduce the risk of neutropenic infections. Afterward, it is possible to manipulate, cryopreserve and store the HSP graft, until HSPs infusion [20]. 

The selection of immune cells consists of removing them from the graft product through a positive selection of hematopoietic stem and precursor cells that selectively express CD34+ antigen on the surface. Several clinical-scale devices are utilized to selectively deplete the immune cells using immunomagnetic separation. After the incubation of the cells in the HSPs graft with an antibody to CD34 covalently bound to a paramagnetic bead, those cells that express CD34 are selectively retained while the remaining cells (including lymphocytes and monocytes) are wiped out thanks to the passage of the cell suspension through a column in a magnetic field [19]. 

### 5.3. Conditioning Regimen

The conditioning regimen aims to systematically deplete the autoreactive immune cells from the peripheral blood, lymphoid tissue, bone marrow and the CNS. The kind of regimen used may vary regionally [4,21]. 

The European Society for Blood and Marrow Transplantation (EBMT) guidelines classified the conditioning regimens in three grades of intensity (high-, intermediate- and low-intensity protocols) based on the degree of myelotoxicity and immunodepletion. The high-intensity protocol includes total body irradiation (TBI) or busulfan combined with Cy (Bu-Cy), each of which is possibly associated with anti-thymocyte globulin (ATG). Doses used for TBI ranged between 12 and 15 Gy, given in 8 to 12 fractions over 4 days, with two to three treatments daily. The American Association of Physicists in Medicine (AAPM) recommends the use of <20 cGy/min in order to reduce the risk of complications [22]. Doses higher than 15 Gy were shown to decrease the relapse rate while increasing the incidence of graft disease and mortality. Low-dose TBI consisting of 2 to 8 Gy given in one to four fractions, in combination with chemotherapy, was demonstrated to be an effective conditioning regimen for HSCT in patients who cannot tolerate myeloablation [23]. 

The intermediate intensity protocol can be further divided into myeloablative or non-myeloablative. In the myeloablative protocol, HSPs are reinfused in order to recover from the aplastic phase. It may consist of a BEAM protocol that includes bischloro-ethyl-nitrosourea (BCNU) 300 mg/m^2^, cytosinearabinoside 200/800 mg/m^2^, etoposide 200/800 mg/m^2^ and melphalan 140 mg/m^2^ associated with ATG. In the non-myeloablative protocol, consisting of Cy 200 mg/m^2^ and ATG, the recovery of hematopoiesis is usually spontaneous and the reinfusion of HSPs is able to reduce the aplastic phase and the related risks. Finally, the low-intensity protocol includes chemotherapy only (e.g., Cy 100 mg/kg) and is not associated with serotherapy [24]. 

### 5.4. HPSs Reinfusion

Following the conditioning, HSPs are reinfused that is usually with hydration, antiemetics and other supportive care measures that are aimed to contain the adverse events of the dimethyl-sulfoxide and cytolysis debris. The engraftment typically occurs on average within 10–15 days [17,24]. 

The stem cell infusion (also called “day 0” of the new immune system) is a relatively simple procedure that is usually performed at the bedside, followed by the administration of supportive treatments and anti-microbial prophylaxis during the aplastic phase and the recovery. Between 10–14 days after the procedure, pancytopenia usually requires irradiated red cell and platelet transfusions, while menstrual bleeding could be prevented by the use of hormonal therapy. Prophylactic and therapeutic anti-infective agents (antibacterial, antiviral and antifungal) are also used in order to cover immunosuppressed periods. 

The grafted cells settle in the bone marrow and start replacing the blood cells in about 2 weeks. The complete immune reconstitution and patient systemic and neurological restoration usually take 12 months or longer [17].

## 6. Efficacy of AHSCT for Multiple Sclerosis 

The main findings from the most relevant studies about the use of AHSCT in MS are presented in Table 2.

### 6.1. Relapses

Our monocentric experience is in line with several meta-analyses that provided evidence that AHSCT can induce long-term remissions for MS patients and confirmed the efficacy of HSCT against MS relapses [39,40]. Indeed, the current literature shows that AHSCT is able to induce complete remission of clinical relapses in 70–100% of treated relapsing-remitting MS (RRMS) patients. In particular, the relapse-free survival rates ranged from 80% at 4 years to 100% at a median of 6.7 years of follow-up [27,32,34,35,41]. This massive anti-inflammatory effect was observed in MS patients with highly active disease prior to AHSCT, as shown by the median/mean annualized relapse rate (ARR) in the previous year ranging from 1.2 to 8 [27,32,34]. In another study, high-intensity AHSCT consistently reduced the ARR from 167 relapses over 146 patient-years before the AHSCT procedure to zero over 179 patient-years at the follow-up [27]. 

Other studies showed that the ARR following AHSCT was markedly reduced, ranging from zero to values below 0.05 [27,30,34]; the ARRs reported by the pivotal trials of highly effective DMTs, although lower than the pre-treatment values, were considerably higher than those reported in AHSCT studies, which ranged between 0.16 and 0.34 [42,43,44,45]. However, such results should be considered with caution [30,46].

### 6.2. Disability

Several studies demonstrated that AHSCT is able to achieve progression-free survival values of up to 100% at 10 years, with a reduction of long-term disability progression in at least 40% of patients [34]. In particular, in RRMS patients, the progression-free survival ranged from 73% to 100% at 5 years, which was maintained for up to 10 years in one study [9,33,34,35]. In another recent cohort where RRMS patients were treated with a BEAM+ATG conditioning protocol, disability-worsening-free survival was reached in 85.5% (range 76.9–94.1%) at 5 years and in 71.3% (range 57.8–84.8%) of patients at 10 years [36]. 

A considerably lower progression-free survival was found in another study showing that 43% of the seven RRMS patients enrolled were progression-free at 5 years [46]. However, the authors assumed that this result was due to the higher disability of the patients at baseline (the Expanded Disability Status Scale (EDSS) ranged between 6 and 7); thus, it could be hypothesized that these patients might have entered the secondary progressive (SPMS) phase early after the enrolment [46]. 

Studies evaluating AHSCT in progressive MS patients showed progression-free survival values ranging from 33% at 5 years to 78.7% at 8 years [9,31]. In a recent study that included 26 SPMS patients with moderate–severe disability, mostly exhibiting signs of clinical or MRI inflammatory activity at the time of the AHSCT, the progression-free survival at years 5 and 10 was 42% and 30%, respectively [37]. 

In a retrospective study of 120 MS patients (52% with progressive MS (secondary or primary)) treated with AHSCT, 90% at 2 years and 85% at 4 years showed no new MRI lesions, with a progression-free survival of about 75% at 2 years and 65% at 4 years [35]. In a recent Italian study, disability-worsening-free survival was 71.0% (range 59.4–82.6%) and 57.2% (range 41.8–72.7%) at 5 and 10 years [36].

However, AHSCT is not recommended by EBMT guidelines in primary progressive MS (PPMS) since registry studies did not demonstrate acceptable levels of effectiveness [21,24].

Confirmed disability improvement was investigated in several studies [30,33,34]. Particularly, it was more frequently reported in RRMS patients and predominantly within the first year after AHSCT. Moreover, a study showed a reduction of at least 1 EDSS point at 1 year in about 50% of patients, and this improvement was constant up to 5 years of follow-up [33]. Another study of 111 RRMS patients demonstrated a significant improvement in EDSS after transplantation [9]. In particular, during the 12 months before AHSCT, the mean EDSS score was increased by 0.94 points; in contrast, during the 12 months after AHSCT, a mean decrease of 0.32 points was observed. Interestingly, although the improvement in EDSS between the pre- and post-treatment periods was higher in relapsing MS patients, a significant change was also found in progressive MS [9]. In addition, the reduction in the median EDSS was also confirmed in other studies showing a change ranging from −0.75 (range −7 to 1.5) to −3.0 points, with a more pronounced decrease in RRMS [30,34]. 

Furthermore, Neurological Rating Scale (NRS) and Multiple Sclerosis Functional Composite (MSFC) scores were found to be significantly improved in patients who underwent AHSCT in several reports [28,32]. 

Several studies investigating the effect of AHSCT on quality of life reported an improvement in both physical and mental health domains in all MS phenotypes, even if it was more marked in RRMS patients [29,31,32]. In the Canadian AHSCT study of 23 patients who underwent AHSCT, the median Fatigue Impact Scale (FIS) score showed a decrease of 36%, with four patients having a 100% reduction [47].

Finally, after AHSCT, 37% of patients in another study improved their functional capacity, where they were able to return to daily life, including returning to work or school [27].

### 6.3. MRI Activity

The intermediate-intensity conditioning AHSCT was able to induce a consistent reduction in the gadolinium-enhanced (Gd^+^) lesions up to 99% compared with the pre-treatment count using a triple dose of gadolinium (Gd) [35]. Similarly, in another study that analyzed 327 MRI scans of 23 patients treated with a high-intensity AHSCT regimen, no signs of MRI activity were detected in comparison with the high disease activity documented prior to AHSCT [27]. Moreover, in a study using the BEAM/ATG regimen, the mean pre-treatment Gd^+^ lesion count was reduced by over 90%, with a consistent decrease from 1.32 to 0.07 after 6 months and to 0.08 at the last follow-up [29]. Other reports demonstrated a reduction in the median T2 lesion volume of 33% after 27 months and of 2.8% after 6 months and at the last follow-up [29,32]. 

In contrast, in another study of seven patients who received a low-intensity regimen, the mean number of Gd^+^ lesions per month and per patient fell from 4.1 before treatment to 0.2 after 3 years using a triple dose of Gd [46]. In this study, the complete remission of MRI activity was achieved in only one patient, suggesting a milder effect on inflammatory lesion activity following a low-intensity conditioning regimen [46]. 

### 6.4. Disease Activity Free Survival

No evidence of disease activity (NEDA-3) was defined as the absence of relapse, EDSS progression and MRI activity (new T2 lesions and Gd^+^ lesions) [48]. According to the current literature, over 60% of patients who underwent AHSCT showed NEDA at 3 years, reaching 70% at 10 years in only one study enrolling a small cohort of RRMS patients [34]. In contrast to these studies, another report showed that, out of the seven patients enrolled, one experienced relapse, four experienced disease progression and six experienced MRI activity at 5 years of follow-up [46]. However, it should be noted that the use of a triple dose of Gd may have potentiated the detection of MRI activity in this study, as it is usually able to detect 75% more lesions than the standard dose [46,49]. In another recent Italian study of 122 RRMS patients treated with the BEAM/ATG conditioning protocol, the risk of NEDA-3 failure was markedly reduced (HR = 0.27 [0.14–0.50], *p* < 0.001) [36].

Furthermore, a recent meta-analysis showed that patients treated with AHSCT achieved higher values of NEDA compared with those treated with currently approved DMTs [50]. Similarly, in a monocentric registry-based cohort study, significantly more patients reached NEDA in the AHSCT group (*p* < 0.05) compared with the alemtuzumab-treated group [51].

### 6.5. Brain Atrophy

It is known that MS patients exhibit higher rates of brain volume loss (BVL) than in healthy controls; moreover, brain atrophy correlates with long-term disability and cognitive decline [52]. 

Different imaging techniques were developed in order to evaluate the BVL between two time points, and several corresponding pathological cut-offs have recently been introduced [53,54,55]. Particularly, yearly values of BVL were −0.49 ± 0.65% and −0.64 ± 0.68% for RRMS and SPMS, respectively, in a large cohort of untreated MS patients [56]. 

Several studies adopting high-intensity regimens for AHSCT showed that, in the short term, the rates of BVL decreased to −3.2% over a median time of 2.4 months, with −15.1% per year and of −2.33% from month 1 to month 12 of follow-up [57,58]. Long-term analysis of the HALT-MS (High-Dose Immunosuppression and Autologous Transplantation for Multiple Sclerosis) study demonstrated that the decrease in brain volume predominantly occurred within 6 months; then, this rate slowed after 3 years and became stable until 5 years after AHSCT [28,59]. Furthermore, another study confirmed the high rates of BVL during the first 6 months after Bu-Cy AHSCT, followed by a stabilization beyond 24 months with −0.32 ± 0.67% by reaching similar values to normal aging healthy individuals [27]. 

In order to provide an explanation about the high rates of BVL reported within the first months after AHSCT, several mechanisms were suggested. Each of these hypotheses, which are not mutually exclusive, may contribute to a different degree to the BVL, relying on individual patients’ characteristics and the treatment agents for the regimen. First, it is possible that the brain atrophy observed in the short term could be due to the remission of inflammation within the brain parenchyma that, in turn, reduces the tissue edema (so-called “pseudo-atrophy”). Second, the ongoing reduction of brain tissue could be already present at baseline and potentially accelerated by neurotoxicity of the AHSCT regimen [37]. More recently, a study of 19 patients, with 12 RRMS and 7 SPMS, treated with high-intensity AHSCT proposed a four-component model [60]. According to the authors, both neurotoxicity and sustained degeneration of damaged brain tissue might contribute to the initial acceleration of BVL [60]. In summary, although the BVL rates detected within the first months after AHSCT were higher than those found in untreated MS patients, they slowed down after a period of 2–3 years, achieving values similar to the healthy population. Thus, this may suggest that effective remission of inflammation could reduce the BVL rates to normal value, at least in selected patients [60]. 

Variations of the lateral ventricular volume, a validated marker of BVL, were estimated in 24 patients treated with BEAM-AHSCT, showing in 75% of them (either RR- or SPMS) that the annualized values were below the pathological threshold [29]. Furthermore, changes in normalized corpus callosum area were comparable to pre-treatment values within 2.5 years after AHSCT, reducing to zero thereafter; at up to 10 years of follow-up, only two of ten patients showed ongoing atrophy [34]. 

## 7. Safety of AHSCT in Multiple Sclerosis

The main AHSCT-related complications in MS are illustrated in Table 3.

### 7.1. Early Complications

The AHSCT procedure includes the administration of cytotoxic chemotherapy that may cause several common adverse effects (AEs), such as anaphylactic reactions, neutropenic fever, infections and sepsis, gastrointestinal toxicity and viral reactivations (particularly Epstein–Barr virus (EBV), cytomegalovirus, varicella zoster virus, etc.) [32,61,62].

Neutropenic fever and/or sepsis require immediate assessment, clinical examination and a prompt therapeutic approach with antibiotics; in case this condition significantly persists even beyond the conditioning phase, it should also be evaluated whether it could be related to the effect of AHSCT. Corticosteroids and paracetamol may help to prevent prolonged pyrexia when the presence of infections is excluded [32]. Moreover, peri-transplant-sustained pyrexia, aside from being a possible infective cause, correlated with poor long-term neurological recovery [32]. Indeed, patients who underwent AHSCT may experience a transient neurological worsening; in particular, fever secondary to the AHSCT and/or to infection may worsen neurological symptoms, such as spasticity, pain, urinary symptoms, weakness and fatigue. For this reason, therapeutic interventions should be promptly carried out to prevent prolonged pyrexia, whatever might be the cause. 

### 7.2. Late Complications

One of the most common late complications of AHSCT is the reactivation of the varicella zoster virus, possibly secondary to the significant immunosuppression related to the conditioning regimen [27]. 

Among viral reactivations, progressive multifocal leukoencephalopathy (PML), a severely disabling and potentially life-threatening disease caused by the reactivation of John Cunningham virus (JCV), mainly in immunosuppressed patients, has raised concerns in the last decade as a potential adverse event of several DMTs, particularly natalizumab [63,64,65]. According to the current literature, only one case of PML has been reported 11 years after an AHSCT in a patient who had received natalizumab treatment for about 3 years, and thus, it could not be considered as related to the AHSCT [71]. Therefore, to date, no cases of PML associated with AHSCT have been reported so far in MS patients. However, the PML risk could be mitigated by adopting adequate wash-out periods after the discontinuation of highly active DMTs potentially at risk of PML and/or dosing the titer of antibodies against JCV prior to the AHSCT procedure.

Following AHSCT, a higher percentage of MS patients compared to the oncologic setting exhibits secondary autoimmune diseases, such as autoimmune thyroiditis, immune thrombocytopenic purpura (ITP), rheumatoid arthritis, Crohn’s disease and acquired anti-factor VIII inhibitor diseases [9,27,66,72]. In a retrospective analysis of patients from the EBMT and Center for International Blood and Marrow Transplant Research (CIBMTR) databases, the incidence of autoimmune AEs mainly consists of thyroiditis, with 5% over a median follow-up of 6.6 years after AHSCT [9,67]. Higher rates (26% and 23%) were reported in a subgroup of patients who had performed the ex vivo manipulation of the graft and in a cohort who had received a conditioning regimen that included alemtuzumab, respectively [27,32].

However, patients treated with alemtuzumab showed higher rates of autoimmune AEs compared to those receiving AHSCT, with almost half of the patients developing a secondary autoimmune condition at 2 years from the second alemtuzumab course [73].

Long-term potential AEs also include effects on fertility and the occurrence of malignancies. For female patients of childbearing potential who underwent AHSCT, the high risk of premature menopause and infertility represents a relevant concern. The risk is significantly associated with the age at transplant, with patients older than 30 years showing the highest risk, and the intensity of the conditioning regimen [74]. Nevertheless, a retrospective analysis of 324 women treated with AHSCT for autoimmune diseases reported 15 pregnancies, 11 of them terminated with 7 healthy live births [68].

A multicenter study reported on post-AHSCT menstrual resumption in 43 MS female patients showing that 30 (70%) started menstruating again after a mean time of 6.8 months [69].

Moreover, in a phase 2 trial of AHSCT for MS, out of 55 patients enrolled, 33 were women and had undergone AHSCT while being of childbearing age at the time of the AHSCT procedure. In a study reporting the outcomes of four pregnancies that occurred after AHSCT, two of them were carried to term with no maternal or neonatal complications. The remaining were not carried to term due to elective termination. In this study, out of 21 males enrolled, one patient has fathered three children since his AHSCT. No newborn complications were reported [70].

Data about male fertility after AHSCT are scarce; however, AHSCT seems to only minimally affect reproductive functions. A small study that enrolled four males who underwent a procedure with Cy and ATG for autoimmune diseases showed a reduction in testosterone compared with baseline values. However, the levels of testosterone remained within the normal range in three patients [42]. Potential complications on the reproductive functions and the possible adoption of fertility preservation strategies should be extensively discussed with patients who are potentially eligible for AHSCT.

Few studies have described the occurrence of tumors following AHSCT. In a recent retrospective study, malignancies were diagnosed in 3.2% of patients (9 of 281 treated patients) [9]. In this analysis, with the exception of three cases of myelodysplastic syndromes typically associated with exposure to cytotoxic drugs, no organ-specific prevalence was detected [9]. Moreover, in the current literature, six malignancies occurred, where one of them was reported in a patient treated with DMT after AHSCT [32,33,71]. Notably, an increased risk of malignancies was reported in patients who received allogeneic HSCT (allo-HSCT) [75]. Similarly, some studies suggested that treatments received prior to AHSCT might play a role in the global cancer risk as MS patients treated with immunosuppressive drugs were more at risk of malignancies, possible due to a reduction in immunosurveillance [76,77].

The risk of mortality represents the main concern limiting the use of AHSCT for MS treatment. Procedures performed between 1995 and 2000 showed transplant-related mortality of 7.3%, 1.3% between 2001 and 2007 and 0.7% between 2008 and 2016, with a dramatic decline in the last decade, where it progressively reduced to 0.2% (1/439) [78]. Across the studies, overall mortality was 2.0% in 829 MS patients transplanted and 2.8% of 281 treated in two retrospective studies [4,9]. A higher rate (4%) was reported in another study; in this analysis, one of the 24 deaths was attributed to hepatic necrosis following busulfan-related sinusoidal obstruction syndrome. Thereafter, during the study, in order to reduce regimen-related toxicity, the dose and route of administration of busulfan were changed [27]. In most of the remaining studies, the mortality was 0% [25,26,28,29,30,31,32,33,34,46,71]. According to recent meta-analyses, patients with specific clinical and demographic features, such as higher disability at baseline, older age and a non-RRMS course, were associated with higher mortality risk [39,40,79]. Moreover, high intensity of the conditioning regimens was associated more frequently with higher rates of mortality (3.13% for high vs. 0.97% for low-to-intermediate intensity). Interestingly, patients who received AHSCT before 2006 were at higher risk, with a significant chronological improvement in the more recent 5-year epochs from 1994 to 2015, probably thanks to a better selection of eligible patients and improving expertise of transplant centers [79].

### 7.3. COVID-19

In the last two years, coronavirus infectious disease (COVID-19) caused by severe acute respiratory syndrome coronavirus-2 (SARS-CoV-2) has become a pandemic threatening global health, as well as affecting the therapeutic management for MS [80]. During the pandemic, a retrospective Italian study found that most of the currently used DMTs were acceptably safe, bringing out some specific elements of risk concerning the use of anti-CD20 drugs [81]. COVID-19 has a great impact on HSCT activity worldwide; the EBMT has, therefore, developed recommendations for transplant programs and physicians caring for these patients [82]. Patients receiving AHSCT for hematological diseases showed a protracted duration of COVID-19 symptoms and a higher risk of generation of highly mutated viruses. Moreover, several studies demonstrated a higher risk of COVID-19 mortality in patients receiving HSCT; however, older age, the presence of comorbid conditions and the immunosuppression due to both treatments and underlying hematological malignancy may also contribute to a more severe COVID-19 course [83]. Particularly, the mortality rate was 11.5% in patients who were not treated with immunosuppressive drugs at the time of COVID-19 diagnosis, while it rose to 33% in those who were immunosuppressed [84].

In the MS setting, not enough data are available in order to define the risk profile of AHSCT during the COVID-19 pandemic. However, reports from the current literature do not suggest a more severe course and higher mortality rates in MS patients who have received AHSCT and faced SARS-CoV-2 infection [81].

## 8. AHSCT and Vaccination

To the best of our knowledge, no data are available about the effect of AHSCT on vaccination response in MS [85]. However, even considering patients receiving transplants for hematological diseases, the literature is scarce and consensus on the timing of post-hematopoietic stem cell transplantation vaccination is currently lacking. In a recent review, it was reported that patients receiving allo-HSCT had a 12 months post-transplant response to influenza vaccine of over 45% that ranged between 10 and 97% at 7–48 months. The response to pneumococcal vaccination at 3–25 months post transplant was 43–99%, increasing over time. For diphtheria, tetanus, pertussis, poliomyelitis and Hemophilus Influenzae type b vaccinations the response ranged from 26 to 100% after 6–17 months from the transplant. The rate of response after meningococcal vaccination at 12 months post transplant was 65%; whilst the hepatitis B vaccine at 6–23 months produced a response in 40–94%. Similarly, measles, mumps and rubella vaccinations at 41–69 months post transplant showed a response in 19–72%. In general, in patients receiving AHSCT, the responses were slightly higher when compared with allo-HSCT [86].

Initial reports on the antibody response after full SARS-CoV-2 vaccination in hematological patients showed that antibody response rates were lower compared with the healthy population. In a prospective study of the Spanish Hematopoietic Stem Cell Transplantation and Cell Therapy Group (GETH-TC) on a large cohort of patients with different diseases (most of them with onco-hematological conditions), 242 (78%) of patients who received allo-HSCT and 73 (85%) of those who have received AHSCT showed detectable SARS-CoV-2-reactive antibodies [87]. Most patients received mRNA-based vaccines and were vaccinated more than 1 year after transplantation [87].

## 9. Factors Influencing the Success of AHSCT and Choice of Conditioning Regimen

Several baseline characteristics were identified as predictive factors for the AHSCT outcome. Some studies demonstrated that a progressive phenotype was associated with a higher risk of post-AHSCT progression (HR 2.33, 95%CI 1.27–4.28) compared with relapsing MS [9,32,33,88]. On the other hand, short disease duration, younger age at the time of the AHSCT and a lower number of previous DMTs correlated with a positive outcome [9]. This was confirmed by a recent retrospective study in which 20 patients with “aggressive” MS underwent AHSCT as a first therapeutic choice. After a median follow-up of 30 (12–118) months, the median EDSS score markedly reduced from 5.0 (1.5–9.5) at baseline to 2.0 (0–6.5), and no patient exhibited further relapses. MRI showed residual disease activities in three patients during the first 6 months after AHSCT, while no further new or enhancing lesions were detected in subsequent scans [41].

In general, these studies contribute toward providing an outline of the “ideal candidate” profile for the AHSCT, suggesting that younger age, highly inflammatory-active MS, mild disability progression and no multiple treatments failure are distinctive elements of better outcomes in terms of efficacy and safety profile following AHSCT [4].

## 10. Cost Effectiveness

In contrast to ongoing repeated treatment with currently approved DMTs, AHSCT represents a “one-off” treatment, providing therapeutic benefits in carefully selected patients. Several studies reported favorable cost-effectiveness in MS patients receiving AHSCT and showing a sustained response compared with some DMTs [89,90]. Although list prices of MS DMTs, ranging from USD 50,000 to USD 70,000 per year in the USA, are apparently lower, the cost of AHSCT (about USD 120,000) is incurred mainly during the first year with minimal subsequent direct costs [91]. Similarly, a UK study showed that the annual costs of second-line DMTs with a long-term schedule of administration, such as natalizumab, fingolimod and ocrelizumab, range from GBP 14,730 to GBP 19,169. Conversely, a complete course of DMTs with an expected long-standing effect costs about GBP 55,000, in particular, GBP 56,360 for alemtuzumab and GBP 50,157 for a 2-year course of cladribine in a patient with an average weight of 70 kg [92]. Furthermore, in a study of SPMS patients with moderate-to-severe disability receiving AHSCT, with a cost of GBP 30,000, the transplant appeared to be more cost-effective than the mitoxantrone (indirect comparator) in two of three scenarios evaluated [89]. The comparator prevailed on AHSCT only when considering the short duration of the effect (sustained for 5 years only). Thus, it is conceivable that the long-standing effect of AHSCT and decrease in mortality rates may contribute to an acceptable cost-effectiveness balance.

## 11. Discussion

In the last few years, the efficacy and safety of the AHSCT procedure for MS treatment have been widely explored, particularly by open-label studies and randomized clinical trials [25,28,29]. However, no clear conclusions could be drawn, mainly due to the small number of patients included in the studies and the high variability in terms of the clinical characteristics and regimens used for AHSCT.

One of the most important advantages of the AHSCT procedure is the direct effect on the CNS. Indeed, the high efficacy of the AHSCT procedure could rely on the effect on the inflammatory cells in the CNS, which are considered to be the main driver of demyelination and axonal damage. To confirm this, data from studies in an oncological setting showed that, after intravenous administration, busulfan diffuses across the BBB with a cerebrospinal fluid (CSF)/plasma ratio of 0.95 [93]. In contrast, the activity of DMTs for MS is mainly confined to the peripheral immune compartment, as most of them do not cross the blood–brain barrier (BBB). Thus, it is conceivable that the pathological increase in BBB permeability observed in active MS patients may affect the drugs’ penetration into the CNS, even if no data supporting this hypothesis are available. This mechanism may be responsible for the potential high efficacy of AHSCT on disability shown in the majority of the studies given that most of the patients eligible for AHSCT typically exhibit aggressive, highly active MS, with some not responding to the approved DMTs [29,33,39,40].

Considering the protocol regimens (high, intermediate or low), there is no consensus as to which of them is superior. This is because of the lack of studies directly comparing these different HSCT conditioning regimens on MS outcomes and the high heterogeneity of the cohorts, including differences in study design, patient selection, outcome measures and length of the follow-up [40,78]. However, higher intensity regimens seem to be more effective, resulting in more sustained disease control, even with a higher risk of infective complications and systemic toxicity [94].

AHSCT represents a good treatment option in light of the cost-effectiveness analyses. Indeed, highly effective second-line therapies might have a lower price, but healthcare costs and treatment management account for considerable healthcare expenses [95]. As observed in most of the cost-effectiveness analyses for DMTs, treating in the early stage and during the RR phase of the disease results is more cost effective than treatments that are started in already disabled patients [96,97]. Indeed, the disability status heavily impacts health costs due to the production losses, the decrease in work productivity and decline in utility, which are strictly associated with the increase of EDSS [96,97]. To confirm this, a European cost-of-illness study showed that the average yearly cost was about EUR 37,100 in moderately disabled patients and EUR 57,500 in more severely disabled patients [98]. In this scenario, AHSCT could have a potential advantage representing a “front-loading” strategy in which the investment of more resources at the onset of the disease, e.g., with the use of more expensive and more effective DMTs, may reduce long-term costs related to disability and associated complications [91,99].

In light of this, studies comparing the AHSCT with high-efficacy DMTs may help to determine the place of AHSCT in the treatment pathway in patients with RRMS but also add information about the potential cost saving of AHSCT compared with more expensive DMTs. Indeed, few retrospective real-world studies have suggested a higher efficacy of AHSCT, even in terms of maintaining NEDA and cognition improvement compared with alemtuzumab [38,51,100]. However, such randomized clinical trials are not available and, to date, only a multicenter, randomized, phase III trial (STAR-MS) aiming to evaluate whether AHSCT has superior clinical efficacy to highly effective DMT (alemtuzumab or ocrelizumab) is still ongoing [101].

Although the potential risk of long-term AEs and the higher rates of mortality compared with DMTs may potentially reverse these advantages, in the last few years, thanks to the increased center expertise of transplant centers and protocol refinements, these safety concerns have been mitigated by promoting programs for the long-term monitoring of various late transplant-related complications. Indeed, until recently, the AHSCT procedure was performed at transplant centers with strong experience and high expertise, with the healthcare services usually tailored for hemato-oncology patients. In the last two decades, the increasing use of the AHSCT procedure in the MS field has highlighted the need for MS-specific supportive care measures, including multidisciplinary teams, in order to improve the center experience and standard of care.

Furthermore, considering that, due to the higher prevalence of MS in women, many MS patients eligible for AHSCT are at childbearing age, the risk of temporary ovarian/testicular failure and infertility secondary to AHSCT require specialist counseling. Moreover, it should be discussed whether high-efficacy DMTs are potentially associated with short- and long-term toxicities, including PML, and other serious infections, which also lead to significant morbidity and mortality rates [102,103]. Indeed, as the main concern of AHSCT is the mortality risk, patients selected for the procedure that AHSCT should be adequately informed that AHSCT is potentially associated with a risk of mortality of approximately 1% in a hemato-oncologic setting, particularly in patients suffering from myeloma, lymphoma and solid neoplasms.

Recent data about the risk of COVID-19 seem to be encouraging. Moreover, even if initial reports suggested lower antibody response rates after a full SARS-CoV-2 vaccination in patients suffering from hematological conditions, data from the National Registry have shown higher response rates [87]. Furthermore, the COVID-19 pandemic has shown that other powerful DMT, particularly ocrelizumab and rituximab, may compromise the vaccination response in MS patients [104]. Notably, no data are available about the effect of AHSCT for MS on COVID-19 vaccination.

Another challenge is whether AHSCT may be effective even in the progressive forms of MS. In the last two decades, AHSCT was used to treat a large number of patients with progressive MS, demonstrating a reduction in the ARR and the disability progression [9,31,66,94]. Moreover, this limited therapeutic effect must be weighed against the potential higher risk of AEs, especially the fatal ones, that are typically associated with advanced disability. Although PPMS seems to not benefit from this treatment, and thus it is was not recommended in the EBMT guidelines [21,24], recent studies have found that several DMTs, including ocrelizumab and rituximab, are effective in reducing the disability progression of PPMS, suggesting that inflammation may play a crucial role in the progressive phase of the disease [105,106,107,108]. Thus, further randomized clinical trials are needed to assess the effectiveness of AHSCT in both secondary and primary progressive MS.

## 12. Conclusions

In conclusion, AHSCT is evolving as a relatively safe and highly effective therapeutic option, where it is able to induce rapid and sustained remission of disease activity with a significant improvement of disability. In recent years, the increased expertise of transplant centers, the improved patient selection and the proper choice of conditioning regimen has contributed to reducing the AE rates and mitigating safety concerns.

Data from the current literature have clearly delineated the profile of the “ideal” candidate for AHSCT. Indeed, the early stages of the disease, with predominant inflammation and low disability level, represent the optimal “window of therapeutic opportunity” in which to propose this therapeutic option [109]. Nevertheless, due to the rapidly evolving therapeutic scenario, it is common practice to offer AHSCT to transplant patients who failed multiple treatments, some of them with potential carry-over safety problems (e.g., PML for natalizumab). However, even in this setting, AHSCT showed an acceptable efficacy and safety profile, while taking into consideration careful monitoring provisions [29].

Finally, definitive evidence from new and ongoing clinical trials is awaited from ongoing RCTs in order to endorse AHSCT as “standard of care, clinical evidence available” for the treatment of aggressive and refractory relapsing MS patients. 

## Figures and Tables

**Table 1:** Demographical and clinical characteristics of the patients who received AHSCT.

Patient	1	2	3	4	5	6
Age (years)	29	28	29	31	55	43
Sex	Male	Female	Male	Female	Male	Female
Disease duration (months)	138	123	171	132	72	84
MS phenotype	RMS	RMS	RMS	SP	SP	RMS
Prior therapies	Natalizumab, rituximab	Natalizumab	IFN-beta1a s.c., teriflunomide, fingolimod, rituximab	Cyclophosphamide, IFN-beta1a s.c., natalizumab	IFN-beta1a s.c., fingolimod, alemtuzumab (two courses)	Alemtuzumab (three courses)
N of relapses at MS diagnosis	4	2	3	6	3	2
N of relapses in the year before AHSCT	2	5	3	3	1	1
N of relapses after AHSCT	0	0	0	0	0	0
EDSS at onset	4.5	1.5	2.0	4.5	6.5	4.0
EDSS before AHSCT	6.0	3.0	5.5	6.5	7.0	5.0
EDSS 6 months after AHSCT	6.0	2.5	4.0	6.5	7.0	4.0
EDSS 12 months after AHSCT	6.0	2.0	4.0	NA	7.5	NA
N of new/enlarged/Gd-enhanced lesions at MS diagnosis	1	1	1	2	1	2
N of new/enlarged/Gd-enhanced lesions before AHSCT	2	5	0	1	0	1
N of new/enlarged/Gd+-enhanced lesions 6 months after AHSCT	0	0	0	0	0	0
N of new/enlarged/Gd+-enhanced lesions 12 months after AHSCT	0	0	0	NA	0	NA
Date of AHSCT	11/Jan/2021	01/Jul/2019	22/Dec/2020	30/March/2021	22/Jan/2020	01/September/2021
Conditioning regimen	BEAM	BEAM	BEAM	BEAM	BEAM	BEAM
AEs (grade) during and/or after AHSCT	None	None	Fever (mild)	Urinary infection (moderate)	Fever (mild)	None
Previous COVID-19 infection	No	No	No	No	No	No
Date of COVID-19 vaccination (both doses) *	May/June 2021	March 2021	April 2021	June 2021	March/May 2021	March/April 2021
COVID-19 third dose of vaccine *	November 2021	November 2021	December 2021	Not done yet	November 2021	Not done yet
Anti-SARS-CoV-2 antibodies titre (date)	163 BAU/mL (October 2021)	43 BAU/mL (September 2021)	54.2 BAU/mL (October 2021)	219.7 BAU/mL (October 2021)	164.9 BAU/mL (October 2021)	287.3 BAU/mL (September 2021)
Comorbidities	Thyroiditis, ulcerative colitis	None	None	None	Hypertension, spasticity	None

AHSCT: autologous hematopoietic stem cell transplantation; BAU/mL: binding antibody unit/mL; COVID-19: coronavirus disease 2019; IFN: interferon; s.c.: subcutaneously administered; i.m.: intramuscularly administered; EDSS: Expanded Disability Status Scale; Gd: gadolinium; MS: multiple sclerosis, SARS-CoV-2: severe acute respiratory syndrome coronavirus 2. * All patients were vaccinated with BioNTech (Comirnaty), Pfizer^®^.

**Table 2:** Relevant studies about the use of AHSCT in Multiple Sclerosis.

Study, Year	Design	Comparator	Conditioning Regimen	Sample Size	MS Form (%)	Median Age (Range) (Years)	Median MS Duration (Range) Years	Median EDSS at Baseline (Range)	Median Follow-Up (Range)	NEDA or EFS	PFS	MRI	TRM (%)	AEs
Mancardi et al., 2015 [25]	RCT	AHSCT MTX	BEAM/ATG	9 12	RR (22) SP (78) RR (42) P (58)	36 ^a^ (22–46) 35 ^a^ (19–43)	10.5 (5–20) 9.8 (2–23)	6.5 (5.5–6.5) 6.0 (5.5–6.5)	4 years	NR NR	43% at 4y 52% at 4y	Reduced number of new T2 MRI lesions at 4 years compared with MTX	0	Early AEs in 80% of patients of AHSCT
Burt et al., 2019 [26]	RCT	AHSCT Different DMTs	Cy/ATG	52 51	RR (50.5) RR (49.5)	34 (18–54) 36 (19–52)	4.7 (0.7–14) 5.4 (0.7–21.2)	3.0 (1.5–6.5) 3.0 (1.0–6.0)	2 years (1–4)	78.5% at 5y 2.97% at 5y	90.3% at 5y 24.7% at 5y	T2 lesion volume improved in AHSCT	0	No grade 4 AEs; 73 grade 3 AEs in AHSCT
Atkins et al., 2016 [27]	Phase II CT	NA	Bu-Cy/ATG + CD34 selection	12 12	RR (50) SP (50)	34 (24–45)	5.8 (1.3–11.2)	5.0 (3.0–6.0)	6.7 years (3.9–12.7)	70% at 3y	69.6% at 3y	No Gd+ or new T2 lesions on 314 MRI scans	0	8 (33%) of 24 patients had grade 2 AEs, 14 (58%) had grade 1 AEs and 2 patients had grade 3 or 4 AEs
Nash et al., 2017 [28]	Phase II CT	NA	BEAM/ATG 24 + CD34 selection	24	RR	37 (NR)	5 (1–12)	4.5 (3.0–5.5)	62 months (12–72)	69.2% at 5y	91.3% at 5y	T2 lesion volume decreased at 6 months; brain volume decreased at 6 months but subsequently stabilized	0	92 grade 3 AEs 100 grade 4 AEs
Moore et al., 2019 [29]	Phase II CT	NA	BEAM/ATG	35	RR (57) SP (43)	37 (21–55)	6.9 (0.7–21.6)	6.0 (2.0–7.0)	36 months (12–66)	90% at 1y 70% at 3y 75% at 1y 50% at 3y	95% at 1y 88% at 3y 75% at 1y 55% at 3y	Reduction of 2.8% in T2 lesion volume	0	5.7% (1 thyroiditis, 1 microcolitis)
Burman et al., 2014 [30]	Obs, long	NA	BEAM/ATG (85%), Cy/ATG (15%)	48 (safety) 41 (efficacy)	RR (84) P (17)	31 ^a^ (9–52)	6.2 ^a^ (0.3–25)	6.0 (1.0–8.5)	3.9 years	68% at 5y	77% at 5y	No Gd+ in 16 patients; MRI event-free survival: 85%	0	Early AE in almost all patients (46%: bacteremia with fever); late AEs in 7 patients (4%: herpes reactivation)
Shevchenko et al., 2015 [31]	Obs, retro	NA	BCNU + melphalan (61%), mini-BEAM-like (39%)	99	RR (43) P (57)	35 ^a^ (18–54)	5 (0.5–24)	3.5 (1.5–8.5)	4 years	80% at 4y	83.3% at 4y	At 6 months, 14 (of 15 patients) showed no MRI activity.	0	NR
Burt et al., 2015 [32]	Obs, retro	NA	Cy/ATG (85%) Cy/ALM (15%)	145	RR (81) SP (19)	37 (18–60)	5.1 (0.7–22)	4.0 (NR)	2 years (0.5–5)	68% at 4y	87% at 4y	No Gd+ in 61 patients (42%)	0	9.6% (7 thyroiditis, 7 ITP); 1.4% (malignancie: 1 lymphoma, 1 breast cancer)
Muraro et al., 2017 [9]	Obs, retro	NA	High (19%), intermediate (64%), low (17%)	281	RR (16) P (84)	37 (17–65)	6.7 (0–34.4)	6.5 (1.5–9.0)	6.6 years (0.2–16)	NR	46% at 5y	NR	8 (2.8)	9 tumors and 14 new autoimmune diseases
Casanova et al., 2017 [33]	Obs, retro	NA	BEAM/ATG	38 (safety) 31 (efficacy)	RR (74) SP (26)	36.7 ^a^ (SD 9.1)	2.3 (0.3–9.4)	6.5 (2.0–8.5)	8.4 years (2–16)	23% of SP: progression of disability 60% of MS: sustained recovery of disability for 7 years after AHSCT	77.4%	Increased T2 lesions in 2 patients after a median time of 5 years	0	55% had fever, 55% had engraftment syndrome; 3 solid tumors (2 breast carcinomas and 1 cervical intraepithelial neoplasm grade 2).
Tolf et al., 2019 [34]	Obs, retro	NA	BEAM/ATG (90%) Cy/ATG (10%)	10	RR	27 (9–33)	2.3 (0.3–9.4)	6.5 (2.0–8.5)	10 years	70% at 10y	100% at 10y	Annual change in the corpus callosum area was similar before and after HSCT	0	17 CTCAE grade 3 3 CTCAE grade 4; 0
Nicholas et al., 2021 [35]	Case series	NA	BEAM/ATG	120	RR (48) P (52)	32 (26–58)	NR	6.0 (5.5–6.5)	1.8 years	53% at 4y	65% at 4y	NR	3 (2.5)	4.2%
Boffa et al., 2021 [36]	Retro	NA	BEAM/ATG	210	RR (58) PP (42)	34 (28–53) 45 (39–58)	NR	6.0 (1.0–9.0)	6.2 years	85.5% at 5y 71.3% at 10y 71% at 5y 57.2% at 10y	NR	NR	3 (1.4)	5.3%
Mariottini et al., 2021 [37]	Open label, retro	NA	BEAM/ATG	26	SP	37 (27–58)	9 (4–18)	6.0 (4.0–7.5)	99 months (27–222)	NR	48% at 3y 43% at 5y 30% at 10y	At year 1, 55% of patients showed an annualized rate of BVL below the pathological threshold of −0.4%	0	73%: fever; 4%: malignancies
Zhukovsky et al., 2021 [38]	Obs	ALM	Cy ATG	69	69 AHSCT 75 ALM	30 35	6.4 (±5.7) 7.0 (±5.7)	3.0 (2.0–4.0) 2.0 (1.0–2.5)	2.8 (±1.6) 2.9 (±1.1)	88 37	97 82	Freedom from MRI events: 93 of AHSCT and 55 of ALM	0	Early AEs: 48/69 grade 3 in AHSCT; 0 in ALME; late AEs: 1.4 in AHSCT; 6.7% grade 3 in ALM

AE: adverse event; AHSCT: autologous hematopoietic stem cell transplantation; ALM: alemtuzumab; ATG: anti-thymocyte globulin; BCNU: bis-chloro-ethyl-nitrosourea; BEAM: carmustine (BCNU), etoposide, cytosine arabinoside (ARA-C) and melphalan; Bu: busulfan; CT: clinical trial; CTCAE: Common Terminology Criteria for Adverse Events; Cy: cyclophosphamide; DMT: disease-modifying treatment; EDSS: Expanded Disability Status Scale; EFS: event-free survival; Gd+: gadolinium-enhanced lesions; ITP: idiopathic thrombocytopenic purpura; long: longitudinal study; MS: multiple sclerosis; MTX: mitoxantrone; NA: not applicable; NEDA: no evidence of disease activity (no relapses, no disability progression and no magnetic resonance imaging activity); NR: not reported; NS: not standardized; PFS: progression-free survival; obs: observational study; P: progressive form of MS (including progressive-relapsing, secondary progressive and primary progressive MS, when not specified); PP: primary-progressive MS; RCT: randomized clinical trial; retro: retrospective study; RRMS: relapsing-remitting MS; SPMS: secondary-progressive MS; TRM: transplant-related mortality. ^a^ Mean values.

**Table 3:** AHSCT-related complications commonly associated with MS patients.

	MS Risk Factor	Measure to Prevent/Treat
Early AEs		
ATG fever [32,61]	Cytokine release	Steroids, antipyretics
Worsening of neurological symptoms [32,61,62]	Fever	Treat cause of fever
LUTS [61,62]	Neurologic bladder	Antimicrobials Rehydration
Hemorrhagic cystitis [32,61,62]	Neurologic bladder	Urinary catheter, rehydration
EBV/CMV reactivation [32,61,62]	Previous exposure to EBV/CMV	Monitoring of EBV/CMV DNA
Pneumonia [32,61,62]	Muscular weakness, immobility	Antimicrobials, early mobilization
Deep vein thrombosis [61,62]	Immobility	Early mobilization, anticoagulants
Falls [32]	Muscular weakness, dehydration	Physiotherapy, fluid monitoring
Late AEs		
PML [63,64,65]	Positive antibodies against JCV, previous treatment with natalizumab	Wash-out after the withdrawal of natalizumab, JCV testing prior to the AHSCT procedure
Varicella zoster reactivation [27]	Immunosuppression	Antiviral prophylaxis
Secondary autoimmune diseases [9,27,32,66,67,68]	Pretreatment with ALM or ATG	Close follow-up
Premature menopause and infertility [42,68,69,70]	Older age, high intensity of the conditioning regimen	Counseling, adoption of fertility preservation strategies
Malignancies [25,26,28,29,30,31,32,33,34,46,71]	Allogeneic HSCT, previous use of immunosuppressive drugs	Close follow-up

AHSCT: autologous hematopoietic stem cell transplantation, AE: adverse event, ALM: alemtuzumab, ATG: anti-thymocyte globulin, EBV: Epstein–Barr virus, CMV: cytomegalovirus, JCV: John Cunningham virus, LUTS: lower urinary tract symptoms, MS: multiple sclerosis; PML: progressive multifocal leukoencephalopathy.

## Data Availability

Dataset is available under reasonable request.
